# Towards Protecting the Injured Party from Errors and Hallucinations of Artificial Intelligence: A Contemporary Legal Perspective

**DOI:** 10.12688/f1000research.179603.1

**Published:** 2026-04-18

**Authors:** Nagwa Abouhaiba, Saed Ahmed Khouli, Maya Khater

**Affiliations:** 1 College of Law, Abu Dhabi University, Abu Dhabi, United Arab Emirates; 2 College of Law, United Arab Emirates University, Al Ain, United Arab Emirates

**Keywords:** Artificial Intelligence; AI Errors and Hallucinations; UAE Civil Transactions Law; Civil Liability and Technology; Protection of the Injured Party; Digital Harm.

## Abstract

The world has been witnessing a growing and accelerating expansion in the use of artificial intelligence (AI) systems, as leading worldwide technology businesses compete to develop systems and software that can simulate the human mind, produce technological solutions, and accomplish activities that previously needed human mental effort. This has given rise to a host of new and unfamiliar legal challenges, including those connected to errors and ‘hallucinations’ that may produce inaccurate or misleading outputs. This paper examines the technical and practical risks associated with the use of AI, with a focus on the phenomenon of hallucinations that has recently become widely discussed, and its potential effects on users and third parties. This study adopts a comparative, analytical legal approach by analyzing the current legal framework to determine the extent to which the law protects the injured party, assessing the adequacy of existing civil-liability rules, and emphasizing the difficulties associated with proving fault and causation, as well as identifying the responsible party within the complex smart-transactions ecosystems. Accordingly, the objective of this paper is to determine how civil liability arising from the use of AI systems in numerous fields may be attributed, and to identify the future legal implications of such systems in light of the general rules of the UAE Civil Transactions Law and the substance of the EU regulation. Accordingly, the study presents a contemporary legal perspective on conventional culpability to safeguard the harmed person against AI faults and hallucinations. It concludes by proposing innovative legal mechanisms, such as strict (objective) liability, transparency obligations, and compulsory insurance, to compensate the injured party for ‘digital harm’ caused by AI technologies. The research indicates that legal responsibility and technical innovation must be balanced to safeguard the damaged party.

## Introduction

The development of AI models has been accompanied by several challenges, chiefly reflected in experts’ skepticism regarding the difficulty of achieving progress in government-funded AI projects and research due to limited computing capabilities, programming difficulties, and expert systems’ failure to meet expectations; such systems were costly to maintain and lacked the flexibility required to address complex real-world problems ( Abou Adel, 2022; El Baroudy et al., 2025). Interest in AI was renewed in the 1990s and the early twenty-first century as a result of advances in computing, the availability of data, and innovation in algorithms. AI was then deployed across multiple fields, culminating in the revolutionary applications we see today in healthcare, finance, transportation, entertainment, and education ( Qiqieh et al., 2025; Mucci, 2026).

Artificial Intelligence (AI) is a term whose use and circulation has increased rapidly in recent times across diverse forums, in the wake of the scientific, technical, and technological surge witnessed worldwide. AI is an advanced scientific and technical field concerned with designing and developing systems and software capable of simulating the human mind in logical reasoning, learning, decision-making, and inference, with the aim of enhancing efficiency and developing advanced technical solutions that facilitate human life. These features have rendered AI a central and indispensable element in human, scientific, practical, and research fields alike. This rapid technological development has, in turn, imposed complex legal issues relating to responsibility for decisions generated by AI models, with the absence of a clear legislative framework representing one of the most salient challenges. On this basis, many advanced countries, including the United Arab Emirates, began to develop and adopt legislative frameworks and governance principles specifically addressing AI. In 2017, the UAE Government adopted a comprehensive national AI strategy, the ‘UAE Artificial Intelligence Strategy 2031,’ and issued regulatory and ethical charters for AI, while introducing legislative amendments in data protection and digital governance ^*^. Similarly, in 2024, the European Union moved toward issuing the Artificial Intelligence Act (AI Act), regarded as the first legislation to provide a legal definition of an AI system, and comprising a set of rules intended to enhance the trustworthiness of AI in Europe.

### Key characteristics of AI

AI is characterized by numerous features that have contributed to its rapid spread and adoption across various fields. The most significant of which may be summarized as follows:

**Autonomy:** AI’s ability to make decisions and execute complex tasks without human intervention and operating decisions autonomously by determining appropriate means to achieve its objective.

**Simulation:** This characteristic constitutes the foundational basis of AI. The core concept of AI is to simulate the capabilities of the human mind in analysis, comprehension, and problem-solving. AI performs complex functions that typically require direct human intervention to process and execute.

**Self-learning and adaptation:** AI’s capacity to learn and derive lessons is among its most distinguishing features. AI can process information and big data using self-learning algorithms that formulate and test assumptions. The more information AI is exposed to, the more its performance evolves; its ability to detect patterns, infer results, adjust behavior, and autonomously analyze data develops through techniques such as Machine Learning and Deep Learning. (Abou Adel et al., 2024; Alhourani et al., 2026; Qiqieh et al., 2025; Adel, 2022; Abdulrahman, n.d.)

**Prediction and decision-making:** AI enables the processing of vast quantities of data at very high speed, empowering it to predict future outcomes or propose decisions based on statistical and algorithmic inferences.

**Scalability and development:** AI’s flexibility enables expanding its applications by increasing inputs and improving algorithms. This characteristic underscores AI’s dynamic and ever-evolving nature, with capabilities that are variable and constantly growing. ( Norvig, 2010).

**Natural language processing (NLP):** AI uses this capability to understand language details which allows it to produce correct answers that meet user requirements. The feature developed through the use of chatbots and voice-assistant applications which includes the virtual assistant ‘Siri’ created by Apple as its most important demonstration ( Nigam, 2024).

***Errors and hallucinations of AI and their impact on civil liability***

AI systems possess distinct attributes which enable them to operate independently while they learn new information and make future predictions. The implementation of artificial intelligence technology changes the established legal framework because its autonomous systems and self-learning capacities create legal challenges which extend beyond technical matters to affect fundamental tenets of contemporary legal systems ( Qutieshat et al., 2026). The most prominent of these issues include the following:

***First - autonomy (agentic AI)***

The autonomous capabilities of artificial intelligence systems create legal difficulties because the systems provide incorrect information which leads to incorrect final results and dangerous situations that result in harm to people and property. The key legal challenges arising from autonomy include:

- **Legal liability:** The autonomy of AI and its ability to perform tasks independently and without any human intervention raises a fundamental question: who bears the consequences of the system’s decisions, acts, or errors - the manufacturer, the owner, the operator, the programmer, or the user? This feature has driven some to adopt the hypothesis that a robot, as one of the most important AI applications, should acquire legal personality as an electronic or virtual person, such that it would be liable to compensate third parties ( Ben Othman, 2020). In our view, however, this hypothesis is not practically or legally workable; even if an artificial juristic personality could be created for intelligent machines and generative AI systems, such systems do not possess an independent Financial Liability from which compensation could be paid to the injured party.
- **The AI ‘black box’ problem:** It is difficult to detect and remediate the damage caused by AI systems since humans cannot see, forecast, or determine how they process data, assess their algorithms, or make autonomous judgments. Unaddressed injury may lead to increased legal and material damages from autonomous decision-making.
- **Legality:** This issue concerns the extent to which AI systems may be granted powers that affect individuals’ fundamental rights, such as judicial decision-making, legal advice, security matters, and military equipment.

***Second - ‘AI hallucinations’***

Hallucinations are one of the biggest issues facing AI systems, particularly ‘generative AI’ based on deep learning and self-learning. Such systems use mathematical algorithms and complicated internal working processes, making it difficult or impossible to anticipate all outputs or judgments under different conditions. When generative AI algorithms produce outputs that are not grounded in the data on which the system was trained, or when intelligent systems do not follow a recognized or explainable pattern of reasoning, ‘hallucination’ or ‘AI hallucinations’ may occur ^†^.

To understand the causes of AI hallucinations, Professor Novotny explains that such hallucinations are a side effect arising from how generative AI operates: intelligent systems use an algorithm to generate outputs by sampling from probability distributions built from large sets of examples. In essence, that algorithm relies on a form of conjecture in arriving at outputs and results; accordingly, it may also produce incorrect statements or acts ( Nasr, 2025).

The reasons underlying AI hallucinations can be summarized as follows ( Coursera, 2025):

**Limited training data:** Biases in the inputs used to train AI models can lead them to produce biased and inaccurate hallucinations presented as if they were reliable facts.

**Model complexity:** If an AI model is so complex that it lacks constraints limiting the type and quality of outputs it can produce, it may generate outputs that are based primarily on conjecture.

**Data poisoning:** Data poisoning occurs when internet hackers inject incorrect, misleading, or biased data into the training datasets of an AI model, which may create a security issues or lead to a cyberattack.

**Overfitting:** An AI model may successfully predict training data but fail to generalize to fresh data. If an AI system is trained to recognize humans and given many images of people, including groups of people standing next to streetlights, the model may incorrectly learn that streetlights are humans and begin to identify them as people. An experimental test conducted by Stanford University in 2024, involving leading legal-AI platforms across over 200 legal queries, revealed that the +LexisNexis Lexis platform reached a hallucination rate of 17% of cases, while the hallucination rates in Westlaw AI-Assisted Research was 33%. The ‘Ask Practical Law AI’ platform provided an accurate responses to queries in only 18% of cases ( POLANI, 2025).

In short, we consider that the difficulty of predicting AI system outputs due to AI hallucinations is not merely a technical problem; rather, it is a legal problem connected to the rules of civil liability. For this reason, most legal systems tend to impose restrictions on AI outputs and decisions whenever they entail legal or social effects.

***Third - technical errors***

The occurrence of technical errors in AI systems is attributed to one of the following causes:

• Errors in data or input.
• Errors in algorithms.
• Errors in self-learning.

Beyond the technical domain, such errors trigger multiple legal issues, the most important include: the liability issue of identifying the actor responsible for the error; the transparency issue of explaining the automated decision produced by AI on the basis of that error; and the security issue of preventing intrusion and manipulation that may arise from any vulnerabilities emerging from such technical errors ^‡^.

Given the legal issues associated with these technical errors, modern legislations - foremost in the UAE - has embedded two general principles governing AI use: (i) the principle of safety, which aims to ensure that all AI systems comply with the highest safety standards and encourages the modification or removal of high-risk systems; and (ii) the principle of transparency, which calls for a clear understanding of AI and how systems operate and make decisions, thereby contributing to building trust and enhancing responsibility and accountability in the use of these technologies.

### The specificity of harm and liability in the face of AI outputs

The harms addressed by civil liability rules differ from those arising from the use of AI systems, as the latter possess a distinct legal character. This creates a point of difficulty in light of the civil liability rules regulated by the UAE Civil Transactions Law ( UAE Civil Transactions Law, Federal Decree-Law No. (25) of 2025), which attributes damage and fault to a direct human act or to the custody (guardianship) of specific tangible things ( Article 246 and Article 271 of Federal Decree-Law No. (25) of 2025). Today, however, we face harms of a different nature arising from AI outputs, harms that are non-material and intertwined with autonomous capabilities. This reveals a new type of harm that is difficult to subject to the traditional standards for proving fault ( Faqih, 2024), or even identifying its source, especially given the overlap among the roles of the developer, operator, and user ( Surden, 2019).

Comparative trends, especially in Europe, are re-examining civil-liability concepts by expanding strict (objective) liability and linking it to risks associated with use of advanced technologies ^§^, as reflected in legislative proposals regulating AI and liability for its products (European Commission, White Paper on Artificial Intelligence: A European Approach to Excellence and Trust *.*, 2020). These proposals are characterized by taking into account the specificity of digital harm and the need to develop effective legal mechanisms that protect the injured party without stifling technological innovation.

The legal system needs to establish civil liability rules which protect injured parties through the identification of persons who bear responsibility for harmful acts through both contract law and tort law. Most legislation does not recognize an independent legal personality for technical systems. A smart system operating under a contractual agreement creates contractual liability when the debtor fails to fulfill his responsibilities which require him to either produce specific outcomes or show proper safety procedures or necessary information disclosure. When harm happens beyond the contractual connection, tort liability emerges on the basis of fault or liability for things;the AI system operator assumes the role of system custodian because he owns control over the system. The custodian becomes responsible for compensating the damaged party when he fails to inform about system dangers or conceals technical details from the public. Many legal scholars view AI systems as harmful and require tougher liability regulations to balance innovation with injury prevention.

Most jurisdictions do not consider AI systems as having autonomous legal personality thus civil-liability standards require attribution of the harmful action to an actual person or legal entity for contractual and tort liability. In a contractual connection (e.g., an AI system operating agreement) contractual liability exists when someone proves that a party broke an obligation which included safety duties or responsibilities to inform about important details. When harm occurs outside the contractual relationship tort liability arises on the basis of fault or under the UAE Civil Transactions Law on liability for things and equipment under a person’s control custodianship so the operator may be treated as the custodian when control and supervision are established. Thus, failing to warn of system dangers or hiding technical information from consumers may result in responsibility and compensation. Many legal scholars consider AI systems “dangerous things” which require tougher liability regulations to balance innovation with victim protection.

To address these harms, the European Union’s AI law adopted the idea of classifying the risks arising from AI uses into four categories: unacceptable risks, high risks, limited risks, and minimal (or acceptable) risks. This classification yields the following:

**Unacceptable risk:** AI systems that pose unacceptable risks, such as the right to dignity, education, work, privacy, equality, health, safety, and human freedom, are often banned because their severity outweighs their potential benefit. For instance, a school may utilize an AI system to grade pupils depending on their behavior, depriving low-scoring students of one or more of those fundamental rights and vice versa. This is social scoring. This category is illegal and unethical since it violates freedom, privacy, and fundamental rights and discriminates against people.

**High risk:** While this category is dangerous, the EU AI Act did not ban its usage by AI systems. In sensitive sectors involving individuals’ rights, such systems are utilized to screen job candidates’ CVs and provide an automatic acceptance or rejection decision based on prior training. Bias or inaccuracy in the automated decision may reject a qualified person without explanation. The hazard of this issue is its influence on individual rights and unlawful applicant discrimination. Thus, the European legislature required human oversight to ensure openness and bias-free decision-making.

**Limited risk:** This category does not harm human rights, such as when a person is mislead by a company’s website into thinking a human replies to inquiries when they are actually connecting with an AI system. Given the low severity of this risk, the European legislature allowed its usage without control, but required users to be told that they were dealing with an AI system.

**Minimal (or acceptable) risk:** This type is utilized in everyday transactions and does not violate human rights. It is non-binding, thus violating it has no legal consequence, such as when an AI program proposes arranging images on a phone or fixing spelling problems when writing. In such circumstances, the program only assists the user; the user can accept or reject the system’s proposal without legal consequences.

### The inadequacy of civil liability rules in the UAE civil transactions law in addressing errors and hallucinations of AI

***The difficulty of proving fault within AI systems***

The UAE Civil Transactions Law grounds tort liability on its three elements: the harmful act (fault), damage, and causation, as provided by Article 246 of the new UAE Civil Transactions Law No. 25 of 2025, which stipulates: “Any harm caused to another shall obligate the perpetrator, even if non-discerning, to compensate for the damage.” Liability is thus understood as arising from a harmful act attributable to an identified actor (most often a human being). This traditional conception faces a fundamental challenge when applied to AI systems, particularly given the non-recognition of legal personality for AI ^**^. The inadequacy of the Civil Transactions Law in addressing AI errors and hallucinations becomes evident. Technical errors arising from the use of an AI system do not result from direct human behavior capable of being assessed by the “reasonable-person standard”; rather, it may result from complex interaction between AI algorithms and self-learning mechanisms. Consequently, it becomes difficult to determine whether the error stems from the programmer, the user, the system provider, or the system itself, leading to difficulty in identifying the legally responsible actor ( Alarie, Niblett, & Yoon, 2018; Surden, 2019). In this context, some scholars ( Al-Sanhuri, 2018; Al-Sahli, 2022) have grounded liability for harms generated by AI uses on liability for things, as provided in Article 271of Federal Decree-Law No. (25) of 2025 issuing the Civil Transactions Law, under the subsection titled “Liability of the Custodian of Things, Animals, and Buildings,” which provides: “Whoever has under his control things requiring special care to prevent their harm, or mechanical machines, shall be liable for the harm caused by such things or machines, except what cannot be avoided, without prejudice to any special provisions in this regard.” This is on the basis that AI systems fall within the concept of “things.” However, AI may not correspond to the strict notion of a “thing,” ( Al-Sahli, 2022; Marqas, 1995; Shannab, 2012) as it occupies an intermediate position: it rises above a thing but does not reach the status of a natural or juridical person.

### The problem of causation in light of the technical nature of AI

This deficiency becomes even clearer when proving causation, which the law requires for liability. Article 255 of the Civil Transactions Law No. 25 of 2025 provides: “Compensation shall, in all cases, be assessed according to the damage suffered by the injured party and the profit lost, provided that this is a natural consequence of the harmful act.”Accordingly, liability is established only where the damage is a natural consequence of the harmful act. This requirement conflicts with the non-linear and the opacity of some AI systems, known as the “black box” problem, which has been previously explained, making it difficult for the injured party to prove that the damage arose as a direct result of the smart system’s decision. It may become impossible to establish the link between act and outcome, which may negate causation, or render legal causation presumed yet exceedingly complex, thereby practically depriving the injured party of protection ( European Commission, 2022; Pagallo, 2018).

### The impact of the deficiencies of traditional liability elements on protecting the injured party

It follows from the foregoing that the traditional civil liability rules, in their current formulation within the UAE Civil Transactions Law, as well as the rules set out in the Consumer Protection Law, are insufficient or ill-equipped to deal effectively with the risks arising from AI errors and hallucinations ( Issa, 2022). Where the Civil Transactions Law requires proof of fault and causation in their classical sense, this does not align with the technical specificity of AI systems or the nature of their outputs. Thus, despite the actual occurrence of damage, the injured party may be unable to obtain compensation because they cannot meet the burden of proof imposed by the general rules. This reveals a genuine legislative gap: rules originally designed for traditional human acts are being applied to technological risks of an entirely different nature, which may conflict with the principle of compensatory justice, one of the core foundations of civil liability, and ultimately leads to weakened legal protection for the injured party ( European Commission, 2020).

### The need to reframe liability rules in light of AI risks

In view of this deficiency, doctrinal calls are increasing to reconsider the traditional concepts of fault and causation when dealing with AI systems, by adopting more flexible legal models, such as presumed liability or risk-based liability ( Khater et al., 2025). This direction is regarded as a means of achieving a necessary balance between encouraging technological innovation, on the one hand, and ensuring effective protection for the injured party, on the other, without undermining the general rules of the UAE Civil Transactions Law ( Imad et al., 2026).

### Strict liability and the risk theory (Liability accompanies benefit)

There is also a trend toward adopting strict liability, grounded in risk theory, as a legal mechanism to address damages arising from AI systems. This theory is premised on assigning liability to the person who benefits from the hazardous activity they have created, without requiring proof of a harmful act (fault). It is rooted in the principle of “liability accompanies benefit”, meaning that whoever derives benefit from a given activity must bear the consequences of its risks. When applied to AI uses, this concept implies that entities operating intelligent systems or deriving benefit from them would bear responsibility for compensating resulting damages ( Pagallo, 2018; Surden, 2019). However, this theory encounters a significant difficulty: the multiplicity of actors involved in the design, operation, and distribution of AI systems, from the manufacturer to the programmer, then the developer, to the supplier, the authorized representative, or even the distributor. Identifying the producer of an AI-related system is therefore difficult and surrounded by ambiguity and complexity ( Faqih, 2024).

Given the European Commission’s conviction that traditional liability rules are insufficient to address AI risks, it adopted the idea of easing the injured party’s burden of proof with respect to AI liability, alongside establishing legal presumptions that assume causation where technical or organizational obligations imposed on smart-system operators are breached ( European Commission, 2022).

### The proposed legal vision for protecting the injured party from errors and hallucinations of AI systems

In light of the inadequacy of civil liability rules to confront the risks of AI systems, including errors and hallucinations, it has become necessary to seek a legal solution to protect society from these risks, by moving from total reliance on traditional civil liability rules to adopting an innovative, flexible, and comprehensive legal vision that ensures full protection and compensation for those dealing with AI systems, in a way that fulfills one of the most important objectives of the UAE Civil Transactions Law, which is to compensate for the damage and prevent harm to others ( El-Erian et al., 2026; Nggilu et al., 2025). This requires considering the establishment of a set of preventive legal obligations that achieve a balance between encouraging innovation and, at the same time, considering the more important requirement of protecting the injured party. This solution proposes a legal vision based on strengthening the duty of transparency combined with the duty of warning, to reduce the risks associated with the use of AI: **“Towards a flexible legislative framework that combines preventive obligations and compensatory liability,”** as follows:

***Transparency obligation for self-operating systems***

This study believes that AI-powered technical systems must be transparent, as transparency is one of the most significant legal requirements to safeguard the harmed party from errors or hallucinations. Transparency helps the injured party and judicial authorities understand how the system operates, its correctness, and its hazards as much as feasible. This requirement follows the UAE Civil Transactions Law’s principles of good faith and non-harm. Transparency lets the judge assess fault and deduce it from the totality of facts and indicia, as well as identify the level of automated behavior’s causation and damage. Since concealing crucial information that prevents evaluation of the intelligent system’s detrimental behavior is proof of error, developers or operators are accountable if such opacity causes actual injury to others ( European Commission,2022; Surden, 2019).

A Chinese court has ruled that a developer is not legally responsible for an AI “hallucination”, establishing a precedent for how such cases may be handled under Chinese law. The ruling, issued by the Hangzhou Internet Court, classifies AI-generated content as a service rather than a product in cases involving hallucinations. As a result, users must prove that a developer was at fault in the content-generation process and that the error caused actual harm. The court said AI-generated content generally does not constitute high-risk activity and that developers have limited ability to control AI responses. Imposing strict liability, it said, could hinder technological innovation ( Qitong, 2026).

***The duty to warn of hallucinations as an independent legal obligation***

UAE legal doctrine and case law require AI system operators to warn of AI-generated hallucinations and output mistakes. AI system operators must warn of the likelihood of hallucinations or inaccuracies in its outputs as part of UAE law’s responsibility of disclosure and warning. Activities with novel dangers, like AI systems, increase its importance. If the developer or operator knows of deceptive or hazardous outcomes, failing to notify users is negligence and civil culpability. UAE courts consider failure of duty to warn a defect that harms others. In sensitive sectors, where technical trust alone is insufficient, unambiguous warnings about the system’s limits and risks are crucial ( Al-Rai et al., 2026; Pagallo, 2018).

***Compulsory insurance for AI systems to compensate the injured party***

Among the elements of the proposed legal vision is the proposal to impose a compulsory insurance system on the use of any high-risk AI systems, to ensure that the injured party obtains compensation without requiring them to bear the burden of proving errors and hallucinations of this complex technology. This proposal is consistent with the logic of guarantee under the UAE Civil Transactions Law, particularly in relation to activities involving special risks, as compulsory insurance contributes to a fair distribution of risk among multiple parties. Developers and users may purchase insurance against damages caused by AI, and this system is considered, in the opinion of jurisprudence, an appropriate solution to cover unexpected damages caused by AI ( Fadel, 2023).

### Compensation funds for persons harmed by AI errors

The idea of establishing compensation funds emerged in several fields, such as compensating victims of traffic accidents where the perpetrator is unknown, and other accidents. Therefore, this idea emerged in the context of compensating persons harmed by AI errors and hallucinations, as a complementary solution to the current civil liability system, especially in cases where identifying the responsible person is difficult or where proving fault and causation is not feasible. Accordingly, compensation funds may be a mechanism that the legislator can adopt in exceptional circumstances, or last resort, to redress harm and compensate injured in a manner that achieves social justice and prevents leaving the injured party without effective protection. Such funds may be financed by developers and users of AI systems.

### The expansion and flexibility of the judge’s authority in assessing technical error and drawing legal presumptions

This view highlights the need to strengthen the civil judge’s role in assessing technical error and extending larger jurisdiction in presuming error or causation if damage is demonstrated. Since AI systems cannot be subjected to the fault standard used to human conduct, expanding the judge’s ability to determine technical error is one of the best ways to address their complexity. Thus, jurisprudence and the judiciary strengthen the judge’s ability to infer fault from facts and indicia, allowing the court to conclude fault or causation if damage is linked to a high-risk technical activity. Given the insufficiency of civil liability rules and the growing interaction with AI systems, this strategy may be the current solution until new legislation regulates AI-related injuries ( Surden, 2019).

### Do we need special legislative intervention or an independent legal model of liability?

In light of the foregoing, and with the increasing harms caused by AI, the question arises as to whether the current reliance on the general rules of civil liability along with corporate policies and AI ethics charters is sufficient to address these problems. The jurisprudence has been echoed between supporters and opponents, while voices calling for the need for special legislation regulating these matters have grown louder. The European experience has clearly confirmed that effective protection for the injured party is achieved only through binding legislative regulation that complements the jurisprudential and judicial roles. Accordingly, society awaits the issuance of a special and independent UAE legislation on the liability of AI, based on both prevention and compensation, in line with the country’s directions toward supporting responsible innovation while protecting individuals’ rights at the same time.

## Conclusion

The study concludes that AI systems, despite the efficiency, speed, and support they provide for decision-making, are simultaneously a source of legal risks that cause harm to their users. While AI applications and systems have delivered significant services across many fields of life, including health, education, law, engineering, security, defense … etc., the world is still facing many legal challenges and issues related to liability, transparency, safety of use, and compensation. and it has been found that these damages are of a new type in nature that cannot be accommodated by the current rules of the Civil Transactions Law. Those rules have been unable to address the harms of AI systems, especially the damages resulting from errors and hallucinations of AI outputs. The study also demonstrates that civil liability rules, despite their flexibility and broad concepts, face real difficulties when applied to AI harms, particularly with respect to proving fault and causation. Accordingly, the study proposes several solutions to avoid these difficulties through a legal vision for protecting the injured party from AI errors and hallucinations. This vision combines preventive obligations, such as transparency and a duty to warn of hallucinations, with effective compensation mechanisms, such as compulsory insurance and compensation funds, together with proposing to expand the judge’s role in assessing technical fault. This achieves a balance between supporting technological innovation and ensuring effective protection for the injured party.

The study concludes with a set of findings and recommendations, including the following:

1. The distinctive characteristics of AI systems themselves constitute a set of legal challenges and issues relating to liability, transparency, safety of use, and security.
2. AI errors and hallucinations represent a real legal risk that may often lead to serious harms and damages, notwithstanding the absence of traditional human fault.
3. The civil liability rules in the UAE Civil Transactions Law, in their current form, even after the issuance of the new Civil Transactions Law No. 25 of 2025, still face practical difficulties in accommodating the technical specificity of smart systems, particularly in proving fault and causation in the field of AI.
4. The UAE judiciary has shown remarkable flexibility in addressing technical harms, by expanding judicial presumptions and assuming fault in high-risk activities.
5. Relaying on AI ethics charters or corporate policies alone is not enough to protect the injured.
6. Comparative approaches, particularly the European experience, confirm the trend toward special or semi-independent legal models of liability for AI, based on easing the burden of proof and ensuring compensation.

### Recommendations

1. Calling for developing a specific UAE legislative framework for AI, or introducing qualitative amendments to the general rules, taking into account the technical nature and newly emerging risks of these systems.
2. Explicitly stipulating preventive obligations that include transparency and the duty to warn against hallucinations, especially in high-risk applications.
3. Adopting a compulsory insurance for AI systems, alongside establishing compensation funds, to ensure redress in cases where identifying the liable party is not possible.
4. The use of AI should be accompanied by a set of laws and charters that ensure optimal benefit to humanity from these systems, while simultaneously developing controls, charters and determinants that contribute to reducing its risks.
5. Supporting and enhancing the role of the UAE judge in assessing technical fault, expand the scope of judicial presumptions, and rely on specialized technical expertise.
6. Benefiting from the European experience in AI liability, while adapting it to the UAE legal environment, in a manner that promotes responsible innovation and protects rights at the same time.

## Ethical considerations

Not applicable.

## Data Availability

No datasets were analyzed or generated during the current study. All sources used in this manuscript are publicly available and are cited in the References section.

## Footnotes

^*^
Among the most important: the UAE Charter for the Development and Use of Artificial Intelligence; the “AI Adoption Guide in Government Services” issued by the UAE Ministry of Cabinet Affairs (2023); and Federal Decree-Law No. (45) of 2021 on the Protection of Personal Data.

^†^
Generative AI hallucinations may take several forms: factual hallucinations (where the AI model produces incorrect information); hallucinated citations and sources (where the model provides fictitious sources to support its claims); contextual hallucinations (where the model misconstrues a context or invents incorrect details); inferential hallucinations (where AI infers incorrect information from the data presented to it); content-expansion hallucinations (where the AI adds unnecessary information or expands beyond the original context); and bias-driven hallucinations (where the AI produces answers reflecting biases present in its training data).

^‡^
Article 18 (1) of the UAE Federal Decree-Law on the Protection of Personal Data provides: “The Data Subject shall have the right to object to decisions issued through automated processing that have legal consequences or significantly affect the Data Subject, including profiling.” Article 18 (4) further provides: “The Controller shall introduce a human element in reviewing automated processing decisions upon the Data Subject’s request.”. The EU AI act, Article 50 (2) provides that providers of AI systems, including general-purpose AI systems that generate synthetic audio, image, video, or text content, must ensure that AI outputs are marked in a machine-readable format and are detectable as artificially generated or manipulated; providers must ensure their technical solutions are effective, compatible, robust, and reliable, to the extent technically feasible.

^§^
The European guidance on robots concluded that the problem of not imposing liability on the robot lies in the current legal framework rather than in the robot itself. Therefore, the prevailing view tends toward the need for a legislative amendment to the UAE Civil Transactions Law to regulate the civil liability of the robot’s designer, operator, and user.

^**^
Regarding giving AI systems legal personality, scholars have differing opinions. One group opposes recognition, arguing that it would amount to the extinction of humanity in favor of AI and that it might absolve potential culpable parties (like the manufacturer, programmer, and developer) of liability for AI-related damages, thereby escalating those damages. Based on resolutions by the European Parliament regarding the significance of recognizing legal personality for AI and robotics, another group believes that AI must be granted legal personality ( Faqih, 2024).
