# Supplementary material for: Towards Protecting the Injured Party from Errors and Hallucinations of Artificial Intelligence: A Contemporary Legal Perspective
Source: F1000Res. 2026 Apr 18;15:569. [Version 1] doi: 10.12688/f1000research.179603.1 (PMC13225102; doi:10.12688/f1000research.179603.1)
Supplement: Peer review history

# Reviewer report: Reviewer response for version 1

**DOI:** 10.5256/f1000research.198134.r476859

**Published:** 2026-04-24

**Authors:** Jamal Barafi

**Affiliations:** 1 University of Sharjah, Sharjah, United Arab Emirates

The manuscript addresses a timely and relevant legal issue concerning liability for artificial intelligence errors and hallucinations, with a particular focus on the UAE legal framework and comparative European approaches. The work is generally well-structured and adopts an appropriate doctrinal and analytical methodology; however, it is only partly clear and accurate in its presentation due to noticeable language weaknesses and grammatical errors. While the study design is appropriate and technically sound for a legal analysis, the explanation of the methodology remains insufficient. The paper does not rely on statistical analysis, which is not required for this type of research, and no underlying datasets are necessary. The conclusions are supported by the analysis. 

**To strengthen the scientific quality of the article, several improvements are recommended:**

**First,** a thorough linguistic revision is necessary to ensure clarity and academic precision.

**Second,** the paper would benefit from incorporating additional relevant legal cases & recent references to reinforce the analytical framework and support the arguments presented.

**Finally, **the author should clearly outline the structure and division of the paper in the introduction to improve readability and logical flow.

  Addressing these points would significantly enhance the rigor, clarity, and applicability of the research.

Is the work clearly and accurately presented and does it cite the current literature?

Yes

If applicable, is the statistical analysis and its interpretation appropriate?

Not applicable

Are all the source data underlying the results available to ensure full reproducibility?

Yes

Is the study design appropriate and is the work technically sound?

Yes

Are the conclusions drawn adequately supported by the results?

Yes

Are sufficient details of methods and analysis provided to allow replication by others?

Partly

Reviewer Expertise:

International human rights law, international organizations law, environmental law, and cybercrime.

I confirm that I have read this submission and believe that I have an appropriate level of expertise to confirm that it is of an acceptable scientific standard.

## References

[1] The Legal Nature of Contracts Concluded by Artificial Intelligence According to The Uae Electronic Transactions and E-Commerce Law No. (46) of 2021 Al-Obeidi, A. , Hussein, S. 2023 24th International Arab Conference on Information Technology Acit 2023Open source preview, 2023.

[2] Robotics and AI Systems: Legal Personality for AI System Under UAE Law and Islamic Jurisprudence Hussein, S. , Al-Obeidi, A. 2023 24th International Arab Conference on Information Technology Acit 2023Open source preview, 2023.

# Response

**Published:** 2026-04-24

**Authors:** Maya Khater

We sincerely thank the reviewer, Dr. Jamal Barafi, for the careful reading of our manuscript and for the constructive and insightful comments. We greatly appreciate the overall positive evaluation of our work.

# Reviewer report: Reviewer response for version 1

**DOI:** 10.5256/f1000research.198134.r476864

**Published:** 2026-04-22

**Authors:** Mohamad Hidayat Muhtar

**Affiliations:** 1 Universitas Negeri Gorontalo, Gorontalo, Indonesia

The article adopts a comparative and analytical legal approach, focusing primarily on the UAE Civil Transactions Law and relevant European legal developments. Its central argument, that traditional civil liability rules are not fully adequate to address AI-related harms because of the difficulties of proving fault, causation, and identifying the responsible actor within complex AI ecosystems, is both relevant and persuasive. The paper also offers several forward-looking legal responses, including strict liability, transparency obligations, compulsory insurance, compensation funds, and a stronger judicial role in assessing technical fault. Overall, the manuscript is relevant, well structured in broad terms, and suitable for publication subject to minor revision.

 The work is generally clear and engages with recent literature, including current legal and policy materials. I answered “Partly” to the first question because the presentation is uneven in some sections, several passages are somewhat repetitive, and a few references appear only loosely connected to the specific issue of AI liability and hallucination-based harm. The paper would be strengthened if the authors relied more consistently on literature directly relevant to civil liability for AI systems, product and technology liability, algorithmic opacity, and evidentiary burdens in civil claims. I answered “Yes” to the question on study design because, for a doctrinal and comparative legal article, the overall design is appropriate and technically sound. The manuscript identifies a clear legal problem and advances a coherent normative discussion.

 I answered “Partly” to the question on methods and analysis because the methodology remains somewhat general. The manuscript states that it adopts a comparative and analytical legal approach, but it would benefit from a clearer explanation of how legal sources were selected, why the UAE and EU frameworks were chosen as the primary comparison, and how the comparative legal analysis was structured. I answered “Not applicable” to the statistical analysis question because no statistical analysis was undertaken, and none is necessary for this type of legal article. I answered “No source data required” because the article is based on publicly available legal materials and secondary sources rather than datasets.

 I also answered “Partly” to the question of whether the conclusions are adequately supported by the results. The article does support its core claim that conventional civil liability rules face difficulty when applied to AI-related harms. However, some of the proposed solutions, particularly compulsory insurance, compensation funds, and broader judicial presumptions, would benefit from being framed more explicitly as normative policy recommendations or supported through somewhat fuller comparative legal reasoning. These are relatively limited points and can be addressed through minor revision. In particular, the authors should clarify the methodology and comparative framework, strengthen the relevance of some cited literature, distinguish more carefully between different liability models such as fault-based liability, strict liability, contractual liability, product liability, and liability for things, and revise the language for greater clarity and academic precision. Subject to these small revisions, I consider the manuscript acceptable for indexing and believe it can make a useful contribution to the literature.

Is the work clearly and accurately presented and does it cite the current literature?

Partly

If applicable, is the statistical analysis and its interpretation appropriate?

Not applicable

Are all the source data underlying the results available to ensure full reproducibility?

No source data required

Is the study design appropriate and is the work technically sound?

Yes

Are the conclusions drawn adequately supported by the results?

Yes

Are sufficient details of methods and analysis provided to allow replication by others?

Yes

Reviewer Expertise:

Law

I confirm that I have read this submission and believe that I have an appropriate level of expertise to confirm that it is of an acceptable scientific standard.

# Response

**Published:** 2026-04-23

**Authors:** Maya Khater

We sincerely thank the reviewer, Mohamad Hidayat Muhtar, for the careful reading of our manuscript and for the constructive and insightful comments. We greatly appreciate the overall positive evaluation of our work.
